# Immunomodulatory factors gene polymorphisms in chronic periodontitis: an overview

**DOI:** 10.1186/s12903-019-0715-7

**Published:** 2019-02-12

**Authors:** Zahra Heidari, Bita Moudi, Hamidreza Mahmoudzadeh-Sagheb

**Affiliations:** 1 Infectious Diseases and Tropical Medicine Research Center, Zahedan University of Medical Sciences, Zahedan, Iran; 2 Department of Histology, School of Medicine, Zahedan University of Medical Sciences, Zahedan, 98167-43175 Iran

**Keywords:** Chronic periodontitis, Cytokine, Gene, Polymorphism, Diagnosis

## Abstract

**Background:**

Chronic periodontitis (CP), defines as destruction of the supporting tissues of the teeth and resorption of the alveolar bone. It is widespread in human populations and represent an important problem for public health. CP results from inflammatory mechanisms created by the interaction between environmental and host genetic factors that confer the individual susceptibility to the disease.

**Aim:**

The aim of the current study was to explore and summarize some functional biomarkers that are associated with CP susceptibility.

**Methods:**

CP is considered to be a multifactorial disease. The pathogenesis of multifactorial diseases is characterized by various biological pathways. The studies revealed that polymorphisms were associated with susceptibility to periodontal diseases. In other word, genetic variations can change the development of CP. However, there are some conflicting results, because there are different variations in frequency of some alleles in any populations. Therefore, we conducted the current review to completely understanding the special biomarkers for CP.

**Results:**

There is some evidence that SNPs in the *IL-1α, IL-1β, IL1RN, IL-6, IL-10, TNF-α, TGF-β1, IFN-γ* and *VDR* may be associated with CP susceptibility.

**Conclusion:**

In conclusion, numerous studies have reported the host genetic factors associated with CP susceptibility and related traits. Therefore, it is prevail to study the multiple SNPs and their effects to find the useful diagnosis methods. The current study will investigate the relationship between polymorphisms in cytokine genes and the susceptibility to the chronic periodontitis.

## Introduction

Accumulation of microbial plaque in gingiva can induce inflammatory responses which lead to periodontal diseases. This inflammation progresses to the periodontitis as a chronic inflammatory condition. In periodontal disease, tooth-supporting tissues are destroyed. In untreated patients, ligamentous support of the teeth is lost. Subsequently, the resorption of the alveolar bone caused loss of the teeth [1–3].

Periodontal disease has two major forms: chronic and aggressive. Aggressive periodontitis is a rapidly progressive condition, but chronic periodontitis (CP) has a relatively slower rate. Chronic periodontitis is common in more than 30% of adults, while up to 13% of the adults will be affected by severe periodontitis [4].

It is believed that CP has multifactorial etiopathogenesis. Host genetic, environmental and microbiological factors are the three major parameters which can determine the natural history of disease [2]. It means that, either subgingival biofilm and host genetic variations together are necessary to cause disease [1]. On the other hand, some strong evidences have been emphasized that host genetic has an important effect in the pathogenesis of periodontitis. In addition, the studies have been conducted on twins reported that the human genome may alter the frequency of the disease in half of the population [4, 5]. In this regard, understanding the genetic basis of CP can be used as an early and a useful diagnostic method for detecting the susceptibility to the disease. Given that, the genes can change the etiology of disease, so we can provide the appropriate treatment methods. It is estimated that there are possibly 20 genes which may increase the relative risk for CP. However, it should be noted that the effects of these genes are not independent of environmental factors and ethnicity [6].

In CP gram-negative anaerobic bacteria induce the attachment loss of tooth from its supporting tissues. This mechanism has been mediated by some regulatory and inflammatory cytokines. In other words, cytokines contribute to periodontitis susceptibility. Therefore, it has been suggested that insufficient production of cytokines against inflammatory activities can cause the disease. Some studies have shown that there was a relationship between cytokine SNPs and CP. Such variations can change the levels of cytokines production, which in turn may result in changes in immune responses and can cause long lasting inflammations. Therefore, study on relationship between genotype and/or allele frequencies and disease susceptibility can introduce some genotypes and alleles that can increase the risk of inflammatory disease [7]. Recently, some scientists have reported the effects of single nucleotide polymorphisms (SNPs) in candidate genes in susceptibility to CP (Table 1.) [2, 8–13]. It seems that the use of the genetic risk score could be useful in detection of the CP susceptibility [14–19]. However, a number of conflicting results have been reported, because there are different variations in frequency of some alleles in any populations. The aim of the current study was to explore and summarize some functional biomarkers such as Interleukin-1 and Tumor necrosis factor alpha; as pro-inflammatory cytokines; IL-6, IL-10; as regulatory cytokines; VDR; as a connective tissue metabolism-associated-gene; that are associated with CP susceptibility.

**Table 1:** Cytokine gene polymorphisms and association with chronic periodontitis

Cytokine gens	SNP analyzed	Associated with periodontitis –/+	Year	Country	Publication
*IL-1α*	*– 889*	*–*	*2013*	*Macedonia*	[24]
	*– 889, + 4854*	*–*	*2015*	*India*	[25]
	*– 889*	*–*	*2015*	*Algeria*	[31]
	*– 889*	*+*	*2000*	*England*	[169]
	*– 889*	*–*	*2003*	*Thailand*	[39]
	*+ 4845*	*–*	*1999*	*America*	[37]
	*+ 4845*	*–*	*2007*	*Japan*	[38]
	*– 889, + 4845*	*+*	*2012*	*America*	[27]
	*– 889*	*+*	*2013*	*China*	[28]
	*– 889*	*+*	*2016*	*China*	[29]
	*– 889*	*+*	*1997*	*America*	[170]
	*– 889*	*+*	*2007*	*Brazil*	[41]
	*– 889*	*+*	*2001*	*Netherlands*	[42]
	*– 889*	*+*	*2007*	*Germany*	[43]
	*– 889*	*–*	*2005*	*America*	[47]
	*– 889*	*–*	*1998*	*America*	[50]
	*– 889*	*–*	*2002*	*Australia*	[51]
	*– 889*	*+*	*2008*	*Germany*	[52]
	*+ 4854*	*–*	*2008*	*Denmark*	[57]
	*– 889*	*+*	*2005*	*England*	[58]
	*+ 4845*	*–*	*2000*	*China*	[61]
	*+ 4845*	*+*	*2000*	*America*	[62]
	*+ 4845*	*+*	*2005*	*India*	[63]
	*+ 4845*	*+*	*2000*	*America*	[65]
	*– 889*	*–*	*2007*	*Finland*	[75]
	*+ 4845* *– 889*	*–* *+*	*2008*	*Greece*	[81]
	*+ 4845*	*–*	*2006*	*Greece*	[121]
	*+ 4845*	*–*	*2009*	*Japan*	[130]
	*– 889*	*+*	*2012*	*China*	[148]
*IL-1β*	*– 511* *+ 3962*	*+* *–*	*2013*	*Macedonia*	[24]
	*– 511* *+ 3954*	*-* *+*	*2015*	*India*	[25]
	*– 511*	*–*	*2015*	*China*	[30]
	*– 511* *+ 3954*	*–* *+*	*1997*	*America*	[170]
	*+ 3954*	*+*	*2015*	*Algeria*	[31]
	*+ 3954*	*+*	*2007*	*Japan*	[38]
	*+ 3954*	*–*	*2003*	*Thailand*	[39]
	*+ 3954*	*+*	*1999*	*America*	[37]
	*+ 3954*	*+*	*2012*	*America*	[27]
	*+ 3954*	*+*	*2016*	*China*	[29]
	*+ 3954*	*+*	*2001*	*Netherlands*	[42]
	*+3954*	*+*	*2007*	*Germany*	[43]
	*–511, – 1464, – 3737, + 3877*	*+*	*2015*	*America*	[44]
	*+ 3954*	*+*	*2015*	*China*	[45]
	*+3954*	*+*	*2005*	*America*	[47]
	*+3954*	*+*	*2005*	*Brazil*	[48]
	*+3954* *– 511*	*+* *–*	*1998*	*America*	[50]
	*+3954*	*+*	*2002*	*Australia*	[51]
	*+3954*	*+*	*2008*	*Germany*	[52]
	*+3954*	*–*	*2016*	*Colombia*	[53]
	*+3954*	*–*	*2015*	*Brazil*	[54]
	*+3954, − 511* *− 31*	*+* *–*	*2015*	*India*	[55]
	*+ 3954*	*+*	*2012*	*India*	[56]
	*+3954, – 511*	*+*	*2008*	*Denmark*	[57]
	*– 511, + 3954*	*–*	*2005*	*England*	[58]
	*+3954*	*–*	*2000*	*China*	[61]
	*+3954*	*+*	*2000*	*America*	[62]
	*+3954*	*+*	*2005*	*India*	[63]
	*+3954*	*+*	*2000*	*America*	[65]
	*+3954*	*–*	*2007*	*Finland*	[75]
	*+3954* *– 511*	*+* *–*	*2008*	*Greece*	[81]
	*+3954*	*–*	*2006*	*Greece*	[121]
	*+3954* *–31*	*+* *–*	*2009*	*Japan*	[130]
*IL1RN*	*IL1R pstl1970, IL1RN mspa111100*	*–*	*2013*	*Macedonia*	[24]
	*IL1RN VNTR*	*–*	*2015*	*India*	[25]
	*IL1RN + 2018*	*–*	*2007*	*Japan*	[38]
	*IL1RN VNTR*	*+*	*2001*	*Netherlands*	[42]
	*IL1RN VNTR*	*–*	*2008*	*Denmark*	[57]
	*IL1RN VNTR*	*+*	*2012*	*China*	[59]
	*IL1RN VNTR*	*+*	*2006*	*Turkey*	[60]
	*+ 2018*	*–*	*2009*	*Japan*	[130]
*IL-6*	*–174*	*+*	*2005*	*England*	[58]
	*–174*	*–*	*2010*	*Turkey*	[68]
	*–174*	*+*	*2007*	*Brazil*	[41]
	*–174*	*+*	*2003*	*Brazil*	[72]
	*–174*	*+*	*2014*	*Brazil*	[73]
	*–174*	*+*	*2006*	*Germany*	[74]
	*–174*	*+*	*2007*	*Finland*	[75]
	*–373* *–597, –572, –190, – 174*	*+* *–*	*2005*	*Japan*	[76]
	*– 174*	*–*	*2009*	*England*	[78]
	*– 572* *– 597, – 174*	*+* *–*	*2004*	*Czech*	[79]
	*–1363* *– 572*	*+* *–*	*2014*	*China*	[80]
	*–174*	*–*	*2008*	*Greece*	[81]
	*–572, – 174*	*–*	*2009*	*Japan*	[130]
	*–174* *+ 874*	*+* *–*	*2006*	*Germany*	[74]
*IL-10*	*– 627, – 1082*	*–*	*2005*	*England*	[58]
	*– 1082, – 819, – 592*	*–*	*2010*	*Turkey*	[68]
	*– 1082*	*–*	*2006*	*Germany*	[74]
	*– 1082*	*–*	*2007*	*Finland*	[75]
	*– 1082*	*+*	*2003*	*Sweden*	[90]
	*– 819, – 592* *– 1087*	*+* *–*	*2004*	*Brazil*	[91]
	*– 592* *– 819*	*+* *–*	*2007*	*Turkey*	[92]
	*– 1082* *– 819, – 592*	*+* *–*	*2012*	*Macedonia*	[93]
	*– 819, – 592* *– 1082*	*+* *–*	*2012*	*China*	[94]
	*– 819, – 592* *– 1082*	*+* *–*	*2012*	*Portugal*	[171]
	*– 1082*	*+*	*2015*	*India*	[96]
	*– 1082, – 592*	*+*	*2012*	*Germany*	[97]
	*– 1082*	*–*	*2015*	*Brazil*	[98]
	*– 1082*	*–*	*2014*	*Brazil*	[99]
	*– 592*	*+*	*2008*	*Brazil*	[102]
	*– 1082, – 819, – 592*	*–*	*2008*	*Germany*	[145]
	*– 1082, – 819, – 592*	*–*	*2001*	*Japan*	[106]
	*– 1082*	*–*	*2006*	*Germany*	[74]
	*– 1082, – 819*	*–*	*2009*	*Japan*	[130]
*TGF-β*	*– 509, – 788*	*–*	*2013*	*Iran*	[10]
	*– 29*	*+*	*2013*	*Iran*	[10]
	*Codons 10 and 25*	*–*	*2010*	*Turkey*	[68]
	*Codon 10* *Codon 25*	*–* *+*	*2006*	*Germany*	[74]
	*– 988, – 800, – 509, Codons 10 and 25*	*–*	*2002*	*Czech*	[129]
	*– 509, + 869, + 915*	*–*	*2009*	*Japan*	[130]
	*+ 915* *Thr263Ile and 713/8delC*	*+* *–*	*2006*	*Turkey*	[131]
	*− 509*	*+*	*2009*	*China*	[133]
	*Codons 10 and 25*	*+*	*2009*	*Macedonia*	[134]
	*Codon 25* *Codon 10*	*+* *–*	*2006*	*Germany*	[74]
*IFN-γ*	*+ 874*	*+*	*2015*	*Iran*	[8]
	*+ 874*	*–*	*2010*	*Turkey*	[68]
	*+874*	*–*	*2006*	*Germany*	[74]
	*+874*	*+*	*2008*	*Germany*	[145]
	*– 5644*	*–*	*2008*	*Iran*	[146]
	*+874*	*–*	*2011*	*Czech*	[147]
*IFN-γR1*	*– 611, + 189, + 95*	*–*	*2015*	*Iran*	[8]
*TNF-α*	*– 308*	*–*	*1997*	*America*	[170]
	*– 1031, – 863, – 857*	*+*	*2003*	*Japan*	[46]
	*– 308*	*+*	*1999*	*America*	[48]
	*– 308*	*–*	*2005*	*England*	[58]
	*– 308*	*+*	*2010*	*Turkey*	[68]
	*– 308*	*–*	*2006*	*Germany*	[74]
	*– 308*	*–*	*2007*	*Finland*	[75]
	*– 308*	*–*	*2008*	*Greece*	[81]
	*– 308*	*–*	*2014*	*Iran*	[108]
	*– 308*	*–*	*2003*	*Czech*	[111]
	*– 1031* *– 857, – 308, – 238*	*+* *–*	*2013*	*China*	[114]
	*– 308, – 863* *– 238*	*+* *–*	*2014*	*China*	[115]
	*– 308*	*+*	*2015*	*Turkey*	[116]
	*– 308, – 238*	*+*	*2008*	*Germany*	[117]
	*– 308*	*–*	*2005*	*Sweden*	[118]
	*– 308*	*–*	*2004*	*Germany*	[119]
	*– 238, – 308, + 252*	*–*	*1998*	*America*	[120]
	*– 308*	*–*	*2006*	*Greece*	[121]
	*– 863, – 857*	*–*	*2009*	*Japan*	[130]
	*– 308*	*–*	*2006*	*Germany*	[74]
*Vitamin D Receptor*	*– 1056*	*+*	*2005*	*England*	[58]
	*FokI* *ApaI, BsmI*	*+* *–*	*2007*	*Japan*	[160]
	*TaqI, ApaI, BsmI*	*–*	*2008*	*Turkey*	[161]
	*TaqI, BsmI*	*+*	*2004*	*Brazil*	[162]
	*TaqI* *FokI*	*+* *–*	*2003*	*Japan*	[163]
	*TaqI*	*–*	*2002*	*China*	[164]
	*TaqI*	*+*	*2013*	*India*	[165]
	*ApaI, BsmI* *TaqI*	*+* *–*	*2013*	*Jordan*	[166]
	*ApaI* *BsmI, FokI*	*+* *–*	*2015*	*Libya*	[167]

## Candidate SNPs involved in CP

### IL-1 gene cluster

Interleukin-1 (IL-1) is a pro-inflammatory cytokine, which encoded by *IL-1* gene cluster at position *2q13–21*. This cytokine produced by monocytes, macrophages and dendritic cells which induce a complex network of proinflammatory cytokines and plays an important role in the regulation of immune and inflammatory responses to infections. IL-1 induces the migration of immune cells to the sites of infection through the expression of adhesion factors on endothelial cells [20, 21].

IL-1 is composed of two different molecules with similar function, IL-1α and IL-1β. IL-1α regulates the intracellular events, whereas IL-1β act as an extracellular protein. It has been reported that the levels of IL-1α, IL-1β and also IL-1/IL-receptor antagonist (RA, anti inflammatory cytokine) have been increased in patients with periodontal diseases [22, 23]. The data showed that the *IL-1* cluster gene SNPs were associated with higher risk for periodontitis [24–26]. Karimbux et al. [27], Mao et al. [28], Yin et al. [29], Zeng et al. [30] in their meta-analysis studies have revealed that *IL-1α* and *IL-1β* genetic variations are significant contributors to CP in different geographical populations. On the other hand, these studies showed that the selected polymorphisms of the *IL-1* genes were associated with susceptibility to aggressive periodontitis but not to chronic periodontitis in the some populations [31]. At the first time, Kornman et al. reported a relationship between *IL-1α − 889* and *IL-1β + 3953* SNPs and disease severity in patients with periodontitis [32]. This study continued by other scientists to examine the relationship between the SNPs in *interleukin-1* gene and periodontitis. Some polymorphisms of the *IL-1* gene have been studied in association with both aggressive and chronic periodontitis: *IL-1α − 889, + 4845; IL-1β − 511, − 31, + 3954* and *IL1RN VNTR* (variable number of tandem repeats), *+ 2018*.

*IL-1α-889C/T* polymorphism is located in the *IL-1α* promoter. *IL-1α − 889* and *IL-1β + 3954* were strongly associated with the periodontitis [32]. This report caused that Kinane et al. evaluated the epidemiological properties of *IL-1* gene polymorphisms in people with periodontitis [33]. Furthermore, it has been shown that *IL-1α − 889* and *IL-1β + 3954* risk alleles could increase and the *IL1RN VNTR* risk allele decrease the IL-1α and IL-1β levels in the gingival crevicular fluid of periodontal patients [34–37]. It means that these SNPs can increase or decrease the gene transcription and protein production levels, which causes changes in the inflammatory responses.

The case-control studies in Caucasians and non-Caucasians have been shown that *IL-1* rare variant (R allele), were different among various populations. In this regard, the carriage rate of the *IL-1α − 889R-allele* varies from 43 to 90% and 35 to 79% in patients and controls, respectively. In addition, Asian populations have a lower carriage rate of the *IL-1α − 889R-allele* (8–23%) than other populations [38, 39]. These findings demonstrate that the ethnicity may affect the carriage rate of SNPs among different population. Therefore, possible positive associations between an SNP and disease within one population may not necessarily be extrapolated to other populations. Only some studies have reported a relationship between the carriage rates of the *IL-1α − 889R-allele* and CP as a single genetic risk factor [40–43].

There are conflicting results about *IL-1β + 3954* SNP. Recently, Wu et al. [44] identified significant associations between moderate to severe adult CP and *IL-1β* polymorphisms in four different ethnicities: Caucasians, African Americans populations, Hispanics and Asians. A meta-analysis by Ma et al. [45], showed that *IL-1β + 3954* polymorphism probably increased the risk of CP in Asians, and might be in association with a strongly increased risk of CP in Indians, but not in Chinese populations. In Asian population the carriage rate of the *IL-1β + 3954R-allele* is lower (≤10%) than the Caucasian populations (13–74%) [38, 39, 46]. Lopez et al., Moreira et al. and Wagner et al. have shown an association between the *IL-1β + 3954R-allele* and CP [43, 47, 48]. Laine et al. reported an association in a sub-group of patients [42]. Galbraith et al. and Gore et al. found a relationship between *IL-1β + 3954R-allele* and periodontitis and disease severity [49, 50]. However, Rogers et al. reported an association between the normal variant (N-allele) of the *IL-1β + 3954* and CP, but not for *IL-1β + 3954R-allele* [51]. In another study, Struch et al. have not found any significant differences for carriage rates of the *IL-1β + 3954R-allele* between CP patients and controls in a Caucasian population [52]. Although some results did not support that the *IL-1β* SNP could be identified as a risk factor for CP, but the synergistic interactions of the some genotypes of *IL-1β* SNPs with environmental factors might play an important role in the pathogenesis of periodontal disease [53, 54].

Amirisetty et al. [55] and Masamattiet al. [56] suggested a strong association of the *IL-1β − 511* and *+ 3954* variants with chronic periodontitis in Indian population. Although, some studies reported carriage rates for *IL-1β-511R-allele*, but, nowadays, *IL-1β-511* polymorphisms haven’t been reported to be associated with CP. The finding showed that the carriage rate of the *IL-1β-511R-allele* was higher among Asians (67%) compared to Caucasians (43–59%) [46, 50, 57, 58].

There were conflicting results about *IL1RN* gene encoding the *IL-1Rα*. There are few studies investigating *IL1RN* gene polymorphisms in periodontitis. The results of some meta-analysis suggested that *IL-1RN VNTR* polymorphism might contribute to an increased risk to CP [59]. In a Turkish population, the frequency of *IL1RN + 2018R-allele* in patients with CP was higher than controls [60]. Results have been shown that the presence of the *IL1RN, IL-1α − 889* and *IL-1β + 3954R-alleles* simultaneously had an association with CP susceptibility [42] and severity [32] in nonsmoking Caucasian patients. In Asian populations, the prevalence of the composite genotype was very low (3%) but in Caucasian populations, it was significantly higher (10 to 46%) [38, 39, 61]. In some case-control studies in Caucasians [47, 62] and non-Caucasians [63], the *IL-1* composite genotype have reported as a risk factor for CP susceptibility. Meisel et al. showed that the *IL-1* composite genotype was associated with periodontal disease only in Caucasian smokers [64]. Moreover, subjects with the *IL-1* composite genotype have increased counts of periodontal pathogens [65]. Nevertheless, the findings revealed a higher frequency of *IL-1* composite genotype in non-smoking patients who had not periodontal pathogens [42]. It means that *IL-1* gene SNPs can change the susceptibility to the disease in the absence of other risk factors.

Taken together, the *IL-1* gene cluster SNPs cannot be considered as risk factors for CP for all populations. However, *IL-1* composite genotype may be a genetic risk factor for Caucasian population.

### Il-6

During an acute and/or chronic infection, immune cells, such as T cells produces IL-6 which causes inflammation in tissues [66, 67].

IL-6 can control the immune responses via the inhibition of type 1 cytokines and activation of type 2 cytokines. In addition, IL-6 involved in regulation of metabolic and neural processes. IL-6 can bind to its membrane glycoprotein GP130 receptor. Subsequently, IL-6-GP130 complex, leading to GP130 homodimer formation and signal induction [66].

The IL-6 is encoded by the *IL-6* gene localized on chromosome *7p21*. Some studies demonstrated that polymorphisms of this gene can affect the concentration of IL-6 in serum [68, 69]. Polymorphisms in the promoter region of the *IL-6* gene affect transcription and expression of IL-6 in individuals. There are three SNPs in the *IL-6* promoter region *(− 597G/A, -572C/G and -174G/C*), that have been reported in chronic inflammations. These SNPs control the up-regulation of IL-6 levels and affect the serum levels of circulating interleukin-6. The *IL-6-174* could influence IL-6 expression levels. The subjects with *IL-6-174C-allele* have lower plasma levels of IL-6 compared to the individuals with *G-allele* [70]. Therefore, *IL-6-174C-allele* may prevent proper immune response against periodontal pathogens in the host.

The carriage rates of the *IL-6-174C-allele* were 37–67% and 44–54% in Brazilian [71–73] and Caucasian populations [58, 74, 75], respectively. In addition, *IL-6 -174*, *− 190* and *− 597* SNPs were not polymorphic in a Japanese population [76]. Heidari et al. [77] Brett et al. [58], Babel et al. [74], Tervonen et al. [75] and Trevilatto et al. [72] have revealed the association between *IL-6-174G/C* and susceptibility to CP. Nevertheless, Nibali et al. found an association for *IL-6-174* in combination With *IL-6 -1480* and *− 1363* SNPs, but not for *IL-6 -174* alone [78].

With regard to the other *IL-6* gene SNPs, it has been revealed that the *IL-6-572* SNP may be a protective factor for CP [79]. In addition, the results have been suggested that the *IL-6-1363 G/T* and *IL-6R + 48,892 A/C* polymorphisms may contribute to genetic susceptibility to CP in Chinese population [80]. However, in a meta-analysis, Nikolopoulos et al. [81] did not show any association between *IL-6-174* and CP, but, it seems that *IL6–174* SNP may be change the susceptibility to CP.

### Il-10

Interleukin-10 (IL-10) is an anti-inflammatory cytokine which has vital role in pathogenesis of periodontal diseases [82, 83]. This cytokine is expressed by various cells specially leukocytes. In addition, T helper 1 (Th1), Th2, CD8 T cells and B cells, from the adaptive immune system can express IL-10. Macrophages, mast cells, natural killer cells in the innate immune system also produce IL-10 cytokine [84]. Interleukin-10 can control viral infections and related tissue damages via stimulating the secretion of immune factors, controlling the phagocytosis and antigen presentation. In addition, IL-10 can play a role in the regulation of proinflammatory cytokines such as IL-1 and TNF-α. On the other hand, IL-10 improves the innate and adaptive immunity [85, 86].

The gene encoded IL-10 is located on chromosome *1q31-q32*. This gene is in a cluster with closely related to the IL-19, IL-20, and IL-24. Polymorphisms in the promoter region of the IL-10 gene can affect the expression of IL-10 cytokine which leads to changes in inflammatory processes [87–89]. There are some conflicting results regarding the association between *IL-10* polymorphisms and CP [90–92]. Some studies to analyze the SNPs in the promoter region of the *IL-10* gene and its relation with periodontal disease have revealed that these polymorphisms might be associated with susceptibility to CP [93–97]. However, in some studies, analysis of allelic and genotypic frequencies of *IL-10* SNPs revealed no causal relationship between the presence of polymorphisms and development of CP [98, 99].

The *IL-10 -1082, − 819*, and *− 592* polymorphisms are in linkage disequilibrium and produce two important haplotypes. The studies revealed that *IL-10 -1082, − 819* and *− 592* polymorphisms were related to the CP in Swedish, Turkish and Brazilian patients [90, 92]. Moreover, in vitro studies have reported that *GCC/GCC* genotype is associated with increased expression of IL-10 cytokine compare to the other genotypes [100, 101].

The in vitro and in vivo studies have been shown that, *IL-10-592R-allele* was related to the lower levels of IL-10 and may change the expression of IL-10 in response to inflammatory disease [100–102]. IL-10 can prevent the periodontal tissue destruction through inhibiting the receptor activator of nuclear factor-κB (RANK) and matrix metalloproteinases (MMPs) [103, 104]. Therefore, subjects with *IL-10-592R-allele* have a higher risk for susceptibility to inflammatory diseases.

With regard to the other *IL-10* SNPs, the results have been shown that *IL-10-1082* polymorphism were not associated with CP susceptibility, especially, in Caucasians, and the carriage rates of the *IL-10-1082R-allele* were 44–81% [91, 105]. However, there was an association between *IL-10-1082 N-allele* and CP in Swedish Caucasians [90].

The *IL-10-819* SNP has been associated with CP in Brazilians but not in other populations [91, 92, 105].

Scarel et al., Sumer et al. and Claudino et al. have found a higher *R-allele* carriage rate for *IL-10-592* SNP in CP patients [91, 92, 102]. The carriage rate of the *IL-10-592R-allele* were 68–75% and 41% - 51 in CP patient and controls, respectively.

Our recent study showed that (unpublished data), the prevalences of AG and GG genotypes of IL-10-1082 in comparison with the AA genotype were significantly higher in CP patients than control groups. In addition, subjects with at least one *IL-10-1082-G* allele significantly had an increased risk for CP. The distribution of the *IL-10-819* and *IL-10-592* genotypes was not different between CP and control subjects. The combination of different genotypes showed that *GCC* haplotype was significantly different between case and control groups. Our results demonstrated that *IL-10-1082* polymorphism was a putative risk factor for CP and associated with increased susceptibility to the disease.

In a Japanese population haplotype frequencies of the *IL-10 -1082, − 819*, and *− 592* SNPs have been analyzed and no significant differences were found between groups in regard to the carriage rates of the haplotypes [106]. The remarkable point was that the *IL-10-1082 N-allele* was absent among the Japanese, but not in Caucasians [90, 106].

Finally, it seems that, *IL-10-592* SNP has been associated with CP susceptibility and might be a genetic marker for CP susceptibility. Given that IL-10 can inhibit the matrix metalloproteinases and reduce the periodontal tissue destruction [103, 104], recommended to reveal the possible relationship between *IL-10* polymorphisms (*− 1082, − 819, − 592*) and chronic periodontitis. These studies need to be replicated to enable conclusions to be drawn.

### TNF-α

Tumor necrosis factor alpha (TNF-α) is a proinflammatory cytokine involved in inflammations. TNF-α can control the production of chemokines or cyclooxygenase products, which consequently induce inflammation. It is produced by many cell types mainly macrophages. TNF-α controls the apoptotic cell death and inflammation and inhibit viral replication through the regulation of immune cells [9, 107, 108]. Insufficient TNF-α production causes various diseases such as cancer, psoriasis and inflammatory bowel diseases [107, 109, 110]. By destructing arachidonic acid, this cytokine causes an increment of prostaglandin E2 concentration, and then activation of osteoclasts. TNF-α stimulates osteoclasts differentiation and along with IL-1 may cause release of the matrix metalloproteinase (MMPs) and destruction of the extracellular matrix and bone resorption [81, 111, 112]. This mechanism probably is done on inflamed periodontal tissues during CP disease.

The human TNF-α gene is located on chromosome *6p21.3*. This gene has an *AU*-rich element in the *3’ UTR* region that is a polymorphic site [9, 108, 113].

It has been shown that the *TNF-α* gene SNPs were risk factors for periodontitis in both Caucasians and non-Caucasians populations. A number of important SNPs in the TNF-α gene have been studied: *− 1031, − 863, − 857, − 376, − 308*, and *− 238* (in promoter region) and *+ 489* (in the first intron).

Yang et al. [114] reported that *TNF-α-1031* SNP was a risk factor for CP. However, there was lack of association between *TNF-α-857C/T* and *-238G/A* gene polymorphisms and susceptibility to CP in Chinese population. Some findings from meta-analysis by Ding et al. [115], supported that *TNF-α-308G/A* and *-863C/A* polymorphisms may contribute to the susceptibility to periodontitis. The results of some studies revealed an association between *TNF-α-308* SNP and periodontitis [116]. The carriage rate of the *TNF-α-308R-allele* in Japanese population [46] was 2–3% and significantly was lower than other populations (18–44%) [49, 58, 75, 111, 117–121]. Our study on an Iranian population did not show a significant difference in frequencies of *TNF-α-308* genotypes and alleles between CP and control groups [108]. Stereological analysis of interdental gingiva in the same CP patients with different *TNF-α-308* SNP also showed no significant differences in volume density of epithelium, connective tissue, collagenous and non-collagenous matrixes and blood vessels between CP patients [9].

With regard to the other SNPs in *TNF-α* gene, the results have been revealed that the frequencies of *TNF-α-238R-allele* were different between various ethnic populations [46, 117, 120]. Soga et al. [46] reported positive associations between *TNF-α − 1031, − 863*, and *− 857* SNPs and CP. But, these findings have not been replicated and needed for further studies to evaluate the real function of the *TNF-α* polymorphisms in CP susceptibility.

### TGF-β

Transforming growth factor beta (TGF-β) is a multifunctional cytokine that controls proliferation, cellular differentiation, apoptosis, angiogenesis, and immune reactions. TGF-β is secreted by various inflammatory cells such as macrophages during tissue injury and regulates synthesis of connective tissue components and matrix proteins by fibroblasts and other cell types via chemotactic mechanisms [11, 122].

This cytokine exists in three isoforms: TGF-β1, TGF-β2 and TGF-β3 that have been expressed in high levels in most tissues. Various cells that secret TGF-β also has autocrine signaling properties because these cells express the TGF-β receptor family. TGF-β can induce differentiation of fibroblasts at the sites of inflammation in order to repair the lesions. In addition, it seems that induction of TGF-β secretion leads to increased eradication of inflammation through apoptotic mechanisms [11, 123].

TGF-β1 is a pleiotrophic growth factor that is involved in the regulation of numerous immunomodulatory processes [124]. It was suggested that TGF-β1 could be considered as a disease predictive biomarker [125]. Studies indicated that the expression level of TGF-β1 mRNA in the regulatory T cells present in the gingival tissue is correlated with periodontitis [126, 127]. Khalaf et al. [128] reported that the expression level of TGF-β1could predict the progression of periodontitis. TGF-β1 can increase osteoprotegerin expression in bone marrow stromal cells. Therefore, it seems that TGF-β1 has a supportive role against the bone destruction process during CP [127].

*TGF-β1–29C/T, -509C/T*, and *-788C/T*, are the main *TGF-β1* SNPs. In our previous study [10], we did not find a significant association between *-509C/T* and *-788C/T* variants of the *TGF-β1* gene and risk of CP in an Iranian population. We proposed that *TGF-β1–29* SNP, may contribute to the development of CP. Moreover, Holla et al. [129], could not find an association between *TGF-β1–29* and *− 509* SNPs and CP in a Czech population. In line with our results, Kobayashi et al. [130] did not find any association between *TGF-β1* polymorphisms and periodontitis in the Japanese population. Also, Atilla et al. [131] have found that *TGF-β-788* variant was not associated with CP in a Turkish population. Nevertheless, the results of our quantitative studies showed that *TGF-β1–509* and *− 29* SNPs were strongly associated with quantitative parameters of connective tissue constituents of interdental gingiva in CP patients [2, 11, 12]. On the other hand, de Souza et al. [132] found that frequency distribution of *TGF-β1–509* genotypes and alleles in the severe CP was significantly different from the control and moderate CP groups. Cui et al. [133] in a meta-analysis suggested that the *TGF-β1–509* SNP was associated with the periodontitis risk in Asians but not in Caucasians. Atanasovska-Stojanovska et al. [134] concluded that polymorphisms of *TGF-β1* gene were associated with an increased risk of CP in a Macedonian population. They found an association between the *-29C/T* SNP and susceptibility to CP.

In regard to the other *TGF-β1* polymorphisms, Babael et al. [135] revealed that the *TGFβ1-codon 25* variant was more common in control subjects than in CP patients. However, Erciyas et al. [68] did not show an association between *TGFβ1-codon 10* and *25* SNPs and periodontitis.

In conclusion, the data suggest that *TGF-β1* gene polymorphisms may contribute to a genetic risk factor for CP. However, further studies analyzing gene-gene and gene-environment interactions are required. Such studies lead to have a better understanding of the association between the *TGF-β1* SNPs and CP risk.

### IFN-γ

Type II class of interferons such as interferon gamma (IFN-γ) play an important role in controlling the immune system. The IFN-γ gene is located on chromosome *12q24* and usually produced by natural killer (NK) cells, Th1 and cytotoxic T lymphocyte cells [8]. IFN-γ can activate the macrophages and increase the expression of Class II MHC molecules and then induce anti-viral and anti-tumor functions. It can inhibit the replication of viruses and inflammatory diseases will occur if the amount of protein change [136]. High levels of IFN-γ are expressed in diseased periodontal tissues and associated with severity of disease. It seems that IFN-γ is involved in the alveolar bone resorption in periodontitis [137, 138].

IFN-γ has a heterodimeric receptor that consists of Interferon gamma receptor 1 (IFNGR1) and Interferon gamma receptor 2 (IFNGR 2). When IFN-γ binds to its receptor, the JAK-STAT signaling pathway is activated. Also, if IFN-γ is attached to the glycosaminoglycan heparan sulfate, it will be deactivated [139].

Polymorphisms of IFN-γ and its receptor can cause chronic inflammatory diseases [8, 140–142]. Recently, Heidari et al. have studied the association between the *IFN-γ* and *IFN-γR1* gene polymorphisms and risk of CP in an Iranian population. They revealed that IFN-γ (+ 874 A/T) was significantly in positive association with chronic periodontitis [8]. In addition, our another investigation (unpublished data), showed that there was a significant relationship between the *IFN-γ (+ 874 T/A)* gene polymorphism and the risk of hepatitis B virus infection as another chronic inflammatory disease in Iranian population. We did not find any significant differences in *IFN-γR1 (− 611A/G), IFN-γR1 (+ 189 T/G), IFN-γR1 (+95C/T)* SNPs between case and control groups.

The *+ 874 T* allele is linked to the *12 C*A repeats and the *A* allele is connected with the non-12 *CA* repeats. This specific sequence provides a binding site for the transcription factor NFkB which induces IFN-γ gene expression [143, 144]. Babel et al., Reichert et al. and Hooshmand et al. studied allele and genotype frequencies of *IFN-γ* polymorphisms in patients with periodontitis [135, 145, 146]. In addition, Reichert et al. [145] confirmed a significant relationship between *IFN-γ + 874* variant and some periodontal pathogens such as *aggregatibacter actinomycetemcomitans* and *prevotella intermedia*. However, Holla et al. [147] did not find significant relationship between variants of the *IFN-γ* and susceptibility to CP or microbial composition. Loo et al. [148] study also did not find any significant differences in their selected genes (*IL-1b, IL-6, IFN-γ* and *IL-10*) between the CP patients and healthy subjects.

In regard to the *IFN-γR1* SNPs, the effects of *IFN-γR1–611* SNP are stronger than of *IFN-γ R1–56* [149]. Rosenzweig et al. [150] found that *G-611* carrier constructs are stronger in promoter activity than constructs carrying *-611A*. *IFN-γ R1 + 95C/*T SNP seems to control the intron-exon splicing process [151].

In conclusion, *IFN-γ* gene seems to be a good candidate for association with CP. However, larger studies are needed to confirm the relationships of *IFN-γ* genetic variations and pathogenesis of CP, because the studies have not yielded any strong indication that it might be an important factor in disease susceptibility.

### Vitamin D

Vitamin D can control the survival and homeostasis of the cell. It is closely related to the immunomodulatory factors which leads to a reduction of the severity of the inflammation. The combination of vitamin D and its receptor (VDR) induces innate immunity systems through inhibiting the Th1 cell functions and activating the Th2 cell responses [152, 153].

Vitamin D and vitamin D receptor are important mediators of bone metabolism. Dysfunction of these compounds and their gene polymorphisms lead to bone resorption, which is one of the most common complications of the periodontal disease. Also, vitamin D and its receptor can regulate phagocytosis by monocytes [154].

The gene encoding VDR is located on chromosome *12q12–q14*. Several studies showed that the *VDR* gene variations were associated with chronic infectious diseases, especially hepatitis and tuberculosis [155–158]. However, till now the actual role of the *VDR* SNPs in CP susceptibility have not been clarified completely. It has been reported that the *Fok1* polymorphism may change the expression of the protein, but not the *Taq1, Bsm1*, and *Apa1* polymorphisms [159]. In this regard, it has been identified that *VDR* SNPs, such as *Taq1, Bsm1, Fok1*, and *Apa1* were associated with CP [58, 160–167]. The carriage rates of the *VDR Taq1R-allele* were 4–23% in Asian populations [163, 164] which were significantly lower than other ethnic populations (42–78%). In some studies, the *Taq1N-allele* has been related to CP susceptibility. There was not an association between *VDR Bsm1* SNP and CP [160–162]. Given that the *VDR* gene can affect both immune functions and bone metabolism, therefore, *VDR* SNPs, specially the *VDR Taq1* may be risk factors for CP susceptibility. Further studies should be undertaken to confirm the current preliminary data.

## Summary and conclusions

Infections can be affected by host genetic factors. The aim of the current review is to provide prognostic genetic markers for detection of the susceptibility of an individual to the chronic periodontitis. Genetic varieties can affect the function of the immune system by changing the transcription of immune factors.

Complex diseases, such as inflammatory bowel disease, type 2 diabetes and many other immune-mediated diseases, share a number of genetic risk variants. In this regard, if a candidate gene has an effect on multiple phenotypes, the pleiotropy occurs. Recently, the effects of pleiotropy in the pathogenesis of complex diseases have been studied [168]. Pleiotropy is associated with many SNPs recorded in the National Human Genome Research Institute (https://www.genome.gov/) brochure. It is interesting to note that in the genes that mediate the immune diseases pleiotropy increases. This definition can improve identifying the risk factors for CP. Therefore, a gene that was recognized as a risk factor for an inflammatory infectious disease might have a similar effect in the process of developing periodontal diseases. For example, IL-10 and 28B, which are important risk factors for hepatitis B virus infection may also be associated with CP. Finally, the overlap of genetic risk factors among diseases may be a useful reason, to analysis such genetic variations in CP. These studies can identify precisely the heritability of CP. As mentioned above, there are a large number of host genetic factors that are believed to be associated with CP susceptibility. It seems that single allelic variants are responsible for disease susceptibility. The most successful example is the identification of SNPs in different cytokine genes in CP.

A genetic variation may be a risk factor for a disease in one population but not for other populations. As the genotype and allele frequencies are vary between different ethnic and geographical populations. With regard to this issue, the studies show that, the *IL-1α − 889, IL-1β + 3954, IL1RN VNTR, IL-6-174, IL-10-1087, TNF-α-308, TGF-β1–29, IFN-γ + 874, VDR TaqI* and *BsmI* SNPs were polymorphic in Caucasian in contrast to Asian populations. It means that, ethnic background can change the association between SNPs and disease susceptibility.

Another important point is the accurate disease phenotype definition. There are two forms of periodontitis, which have some similar clinical symptoms: chronic periodontitis and aggressive periodontitis. Therefore, correct disease classification should be used in patient selection.

The studies with regard to the identifying significant genetic factors for susceptibility to chronic periodontitis are not reproducible always. There are differences among the various studies for some SNPs. Nevertheless, there is some evidence that SNPs in the *IL-1α, IL-1β, IL1RN, IL-6, IL-10, TNF-α, TGF-β1, IFN-γ* and *VDR* may be associated with CP susceptibility. These SNPs can change the signaling pathways of Wnt/b-catenin, p53 and JAK/STAT and induce CP. Such signaling pathways are believed to modulate the host immune system and are considered as useful biomarkers for determining CP.

It should be noted that, genetic predisposing to CP is not the only risk factor for disease. The interaction between genetic, bacterial and lifestyle factors can also affect the disease susceptibility. In other words, individual habits, microorganisms and diet can affect the expression of the gene through the changing the individual’s epigenome.

In conclusion, the study has implied the relationship between genetic variations and CP susceptibility and related traits. However, the real mechanism of interaction between these factors and host immunity functions not specified yet. Moreover, genetic factors alone cannot explain pathogenesis of inflammation. Therefore, further investigation of other host genetic and environment factors, by novel technologies are needed. This process provided therapeutic and preventive strategies for CP.

## Abbreviations

CP: Chronic periodontitis
IFN-γ: Interferon gamma
IL-1: Interleukin-1
IL-10: Interleukin-10
IL-6: Interleukin-6
SNPs: Single nucleotide polymorphisms
TGF-β: Transforming growth factor beta
TNF-α: Tumor necrosis factor alpha
VDR: Vitamin D receptor
